# Electronic Tongues for Inedible Media

**DOI:** 10.3390/s19235113

**Published:** 2019-11-22

**Authors:** Dmitry Kirsanov, Daniel S. Correa, Gabriel Gaal, Antonio Riul, Maria L. Braunger, Flavio M. Shimizu, Osvaldo N. Oliveira, Tao Liang, Hao Wan, Ping Wang, Ekaterina Oleneva, Andrey Legin

**Affiliations:** 1 Institute of Chemistry, St. Petersburg State University, Mendeleev Center, Universitetskaya nab.7/9, 199034 St. Petersburg, Russia; d.kirsanov@gmail.com; 2 Nanotechnology National Laboratory for Agriculture (LNNA), Embrapa Instrumentação, São Carlos, SP 13560-970, Brazil; daniel.correa@embrapa.br; 3 “Gleb Wataghin” Institute of Physics, University of Campinas, Campinas, SP 13083-859, Brazil; gabriel-gaal@hotmail.com (G.G.); malubraunger@yahoo.com.br (M.L.B.); 4 Brazilian Nanotechnology National Laboratory (LNNano), Brazilian Center for Research in Energy and Materials (CNPEM), Campinas, SP 13083-970, Brazil; fmshimizu@yahoo.com.br; 5 São Carlos Institute of Physics, University of São Paulo, São Carlos, SP 13566-590, Brazil; chu@ifsc.usp.br; 6 Biosensor National Special Laboratory, Key Laboratory for Biomedical Engineering of Ministry of Education, Department of Biomedical Engineering, Zhejiang University, Hangzhou 310027, China; cooltao@zju.edu.cn (T.L.); wh1816@zju.edu.cn (H.W.); cnpwang@zju.edu.cn (P.W.); 7 Laboratory of Artificial Sensory Systems, ITMO University, Kronverkskiy pr, 49, 197101 St. Petersburg, Russia; ekaterina.oleneva@inbox.ru

**Keywords:** electronic tongue, multisensor system, MSS, inedible media analysis

## Abstract

“Electronic tongues”, “taste sensors”, and similar devices (further named as “multisensor systems”, or MSS) have been studied and applied mostly for the analysis of edible analytes. This is not surprising, since the MSS development was sometimes inspired by the mainstream idea that they could substitute human gustatory tests. However, the basic principle behind multisensor systems—a combination of an array of cross-sensitive chemical sensors for liquid analysis and a machine learning engine for multivariate data processing—does not imply any limitations on the application of such systems for the analysis of inedible media. This review deals with the numerous MSS applications for the analysis of inedible analytes, among other things, for agricultural and medical purposes.

## 1. Introduction

From the early stage of multisensor systems (MSS) development, considerable experimental efforts were made and many scientific papers were published on “inedible” topics. In recent years, one can speak about the application of taste-sensing systems to the analysis of inedible samples as one of the promising directions of MSS research.

Nevertheless, since various “electronic tongues” and “taste sensors” were primarily suggested and applied for taste and flavor evaluation, it would be reasonable to clearly designate the area of the present review.

First of all, any food applications of MSS are omitted for obvious reasons. The same is true for most fermentation monitoring tasks. Although some of them are related to intermediate process stages (e.g., [1]), in general, the final target of MSS application was different edible products. Similar reasoning is valid for MSS application in pharmaceutics. The vast majority of MSS papers on this topic deal with either taste evaluation of drugs or taste masking; the analysis of inedible products by MSS can be rarely found in this field.

Although most of the media mentioned below are undoubtedly inedible, such as soils or biological samples obtained for medical tasks, it is required to give some specific comments about water analysis. A part of this review is devoted to waste and natural water monitoring, but one should keep in mind that the difference between natural and e.g., potable water can be quite subtle, if there is any difference at all, and some natural waters might be drinkable. Furthermore, the design of MSS applied to the analysis of different types of water can also be very similar. While the analysis of potable water is beyond the scope of this review, the evaluation of undrinkable water properties is an important topic to be addressed, and it will be also discussed below.

Last, only chemical multisensor systems will be observed in this review. Such systems are described as an ensemble of chemical sensors of various compositions with different sensing properties toward simple or complex target analytes. The sensors used in these systems are usually not highly selective to a single analyte and rather show cross-sensitivity to several analytes of similar or different chemical composition. Physical and chemical mechanisms that determine the sensitivity of the sensors might be diverse, although most of the multisensor systems reported so far were based either on electrochemical or optical principles. The output of the multisensor systems can be represented as a matrix with samples in rows and the sensors’ responses in columns. The measured dataset should be processed with appropriate machine learning methods in order to extract meaningful information for further visualization or calculations. Optical systems based on methods similar to conventional spectroscopy of any kind will not be discussed in this paper.

## 2. Water Analysis

Although the original purpose of MSS usage was taste evaluation, they have been also considered as promising tools for environmental monitoring. Traditional methods of water analysis, such as inductively coupled plasma atomic absorption (or emission) spectroscopy (ICP AAS or AES), various versions of liquid chromatography, spectrophotometry, etc., manual “wet” laboratory analysis e.g., chemical oxygen demand (COD) or biochemical oxygen demand (BOD) evaluation, and bioassay tests for water toxicity estimation are well established and reasonably precise. Meanwhile, MSS also have certain advantages, such as rapid, real-time quantitative and qualitative detection of analytes and long-term autonomous monitoring applicability. Taking into account the significantly lower cost of MSS instrumentation and of MSS analysis compared to most existing analytical methods, such multisensor devices are highly promising in this field. The studies devoted to the application of MSS for various tasks of water analysis and discussed in this section are summarized in Table S1 (Supplementary Materials).

The earlier attempts of MSS application to water analysis appeared about 25 years ago. The first studies [2,3,4,5] were concerned with the application of MSS for traditional water analysis, i.e., multicomponent quantification of potentially dangerous substances.

A sensor array comprising 20 solid-state and polymeric sensors was used to analyze 180 multicomponent aqueous solutions containing Cu^2+^, Pb^2+^, Cd^2+^, and Zn^2+^ cations and inorganic anions such as Cl^−^, F^−^, and SO_4_^2−^. Cation concentrations were in the range from 10^−7^ to 10^−5^ mol/L, which has been chosen according to the practice of industrial waste water monitoring, while anion content varied from 10^−4^ to l0^−2^ mol/L for chloride and sulfate and was fixed for fluoride at 10^−5^ mol/L. Three data processing methods were adopted to correlate the sensor array readings with concentrations in the complex solutions: multilinear regression (MLR), partial least squares (PLS), and artificial neural networks (ANN). The best results were achieved using ANN. The mean relative errors (MRE) calculated for the simultaneous determination of all components were within 10%, including the MRE for zinc and sulfate ions; however, the direct sensitivity toward these ions was low and poorly reproducible for all used sensors [2].

A similar MSS was applied to determine the total content of Cu, Cd, Zn, Fe, Cr(VI), chloride, sulfate, and hydrogen ions [3]. One hundred and fifty model mixed solutions were prepared and studied within this research. The measured data set was divided into training, validation, and test sets. Prior to the data processing using machine learning methods, a preliminary qualitative recognition of the analytes was performed using principle component analysis (PCA) and self-organizing map (SOM). Further quantitative calculations were performed by partial least square regression (PLS), non-linear least squares (NLLS), and back-propagation neural net (BPNN) within the data classes obtained during recognition. It was shown that this approach might significantly improve the MSS performance.

A taste sensor was applied in [4] to distinguish pure water samples from model ones contaminated by cyanide and cyanide complex. The recognition of target analytes by MSS was reported; nevertheless, it can be considered only as a proof of concept because no common analytical metrics or figures of merit were presented in this work. It should also be mentioned that it is not necessary to use an MSS for the detection of a single contaminant, as it can be performed by an individual sensor or some traditional analytical method.

A voltammetric MSS was applied in [5] for the monitoring of water processing at a treatment plant. The system comprised four working electrodes: gold, iridium, platinum, and rhodium. Three replicated water samples taken from nine locations across the water treatment plant were measured. PCA was employed for data processing. A possibility to recognize samples with different purification rates was reported that could be used to monitor the water treatment process and to evaluate the effectiveness of the plant filters. The necessity of counteracting the drift of the voltammetric sensors was pointed out.

Along with different types of electrochemical sensors used in various MSS for water analysis, such as the above-mentioned potentiometric [2,3,4] and voltammetric [5] ones, the impedimetric sensors [6] were also applied for this analytical task. Impedance spectroscopy was employed for MSS, e.g., in [6]. It is beneficial to use impedance measurements because in such a case, the materials of the sensing units do not need to be electroactive, and there is no need in a reference electrode, unlike for conventional electrochemical methods. Complex impedance of the whole system was measured over a range of varying frequencies, 20–10^5^ Hz, applied to interdigitated electrodes covered with Langmuir–Blodgett films of stearic acid, a polyaniline oligomer, polypyrrole, and their mixtures. The MSS was successfully applied to distinguish the mineral water samples from two different brands. This work shows that conducting polymers can be used to sense the different capacitance patterns of chemicals presented in a sample under study, and therefore, such sensing materials can be applied for MSS development.

Several MSS based on biosensor arrays and bioelectronic tongues [7,8,9,10] were also developed and studied for water analysis. A novel biosensor chip with two different strains of microorganisms (yeast and bacteria) was proposed in [7]; the strains were immobilized onto four individual Pt electrodes. Such chip design allows the evaluation of BOD and of polycyclic aromatic hydrocarbons (PAH) degrading ability of the bacteria at the same time. The proposed system was applied both for model and real samples (municipal wastewaters). The reported LOD (limit of detection) values were equal to 0.1 mg/L for naphthalene, which was used as model PAH, and 1 mg/L for BOD. This approach provides a great opportunity to use different microbial strains onto the electrodes, adapting the system for a particular analytical task and increasing the amount of information about a sample.

Enzymatically-modified screen-printed amperometric sensors were suggested for the classification of wastewater samples with four treatment quality levels (from untreated to normal) [8]. A sensor array with eight Pt electrodes, modified with four different enzymes, was used in dynamic response curves acquisition mode. The obtained data were preprocessed by the multiplicative sensitivity correction method to eliminate the temporal drift occurred during the measurements. The authors highlight that this step is essential for further multivariate data analysis, showing the graphs of PCA scores for the raw and preprocessed data. Clear recognition of the data classes, consistent with the water quality levels, becomes possible only if the sensor data were corrected before the multivariate data analysis. This study demonstrates the advantage of MSS to be able to perform an integral evaluation of water quality without time-consuming biological tests that are nearly impossible to be used as an alarm indicator in the routine real-time monitoring of water treatment. Unfortunately, this interesting approach was not thoroughly verified, since only a few real water samples were studied.

An MSS combining an array of inhibition biosensors and ANN for data processing was developed [9] for the detection of water contamination by pesticides. The system consisted of three amperometric pesticide biosensors, modified with different acetylcholinesterase (AChE) enzymes, and it was used for the analysis of model aqueous solutions with mixed pesticides and of several real river water samples. The appropriate prediction ability of the MSS was demonstrated; the detected concentration of pesticides was 0.79 nM for dichlorvos and 4.1 nM for carbofuran, respectively. Further, this approach was developed into the so-called bioelectronics tongues that combine biosensors and chemical sensors in a single system [10].

It should be specifically mentioned that the idea of using biosensors in cross-sensitive sensor arrays looks rather contradictory. Biosensors are generally based on biochemical enzymatic reactions that are usually characterized by exceptional specificity and selectivity. In certain cases, a slight cross-sensitivity, e.g., toward a group of similar analytes, might be observed; however, it is not a very typical situation for biochemical reactions. Overall, one should not expect significant cross-sensitivity effects from most of the biosensors.

The ideas about “sensor array” were also exploited with respect to selective chemical sensors, ion-selective electrodes (ISEs). For example, sets of ISEs were applied in [11,12] for the analysis of hydroponic liquids and soil nutrients such as NPK (nitrogen, phosphorous, potassium). The authors of [13] also presented an ISEs array with multivariate data processing of the measurement results for the monitoring of nitrate, nitrite, and ammonium levels in waters. A recent paper [14] describes an array of ISEs for the simultaneous determination of chloride and several metal cations (Ca, Cd, Cu, Pb) in water. PCA was applied as a data compression technique for reducing the complexity of the sensor data. The compressed dataset was further processed by ANN in combination with a novel preprocessing method to improve the ANN prediction accuracy.

It should be noted that the usage of ion-*selective* sensors, as well as biosensors, may oppose the logic of MSS. Nevertheless, since the selectivity of many ISEs can be quite moderate in real complex mixtures, such ISEs are indeed cross-sensitive to some extent. At the same time, there is no doubt that the sensors with enhanced cross-sensitivity should be specifically studied and used in MSS development to conform better with the core principles of multisensor approach.

While optical MSS are frequently used to identify and monitor various chemicals in air, they are rarely applied for the detection of water contaminants. An optical MSS [15] was used for the identification of different toxic chemicals that may be presented in a water distribution network due to emergency situations. Intermediate industrial products, drugs, and pesticides (six compounds in total) were taken to apply the MSS for the identification of various hazardous substances. Although the individual sensing layers demonstrated the limited selectivity toward the chosen chemicals, the optical MSS was sensitive to the mentioned analytes in the range from 10^−7^ mol/L to 10^−4^ mol/L. The specific MSS sensitivity patterns to the different hazardous compounds under study allow their clear identification using the PCA scores graph.

It should be pointed out that MSS can be more versatile in multicomponent quantitative analysis compared to most traditional methods. The majority of instrumental analytical methods are sensitive either to inorganic or to organic components in water. In rare cases when an instrument can sense both types of analytes, a recalibration or elaborated specific tuning of the device would be still necessary. Electrochemical MSS can be sensitive both to inorganic and organic water pollutants providing the comprehensive results. However, this statement is true only if an MSS comprises a sufficient number of highly cross-sensitive sensors (e.g., [16]).

One of the advantages of MSS is a possibility to perform simultaneously both quantitative and qualitative analysis of aqueous media. Certainly, in quantitative water analysis, any MSS would have numerous rivals, such as well-developed laboratory analytical methods (mentioned above, and many others). At the same time, MSS can produce an integral image of global water quality, which is a unique feature of this method. It is not necessary to perform a multicomponent quantitative analysis using an MSS because the integral water chemical image can be produced without explicit knowledge about the concentration of water components. This option emerged about two decades ago, and nowadays, it is one of the most favorable and promising features of MSS application for water analysis as well as for some other tasks [5,16,17,18].

It was suggested in [17] that MSS might be capable of providing integral qualitative images of multicomponent liquids and of functioning in toxic and potentially hazardous media, which is suitable for long-term process monitoring in industry. This “chemical imaging” was demonstrated on a set of natural and tap waters.

An MSS containing the electrodes based on RuO_2_, C, Ag, Ni, Cu, Au, Pt, Al, Sn, Pb, and C (graphite) was used in [18]. The system was used for the classification of different natural and treated waters (seven mineral waters, tap water, and osmotized water). The MSS responses from seven selected electrodes were processed by fuzzy ARTMAP (Adaptive Resonance Theory) neural networks. The optimized system was able to distinguish the mentioned water samples with a misclassification rate of 7%.

A similar voltammetric MSS [19] consisting of eight metal electrodes (Au, Pt, Rh, Ir, Ag, Ni, Co, and Cu) was applied for the recognition of wastewater at the water treatment anaerobic membrane bioreactor and for the quantification of major water parameters routinely measured in the laboratory: COD, BOD, ammonia, phosphate, sulfate, alkalinity, etc. The prediction results for the first four parameters were reasonable, while the total number of calibration (28) and test (10) samples was not large enough to make definitive conclusions about the applicability of the studied MSS for industrial analytical tasks.

Although some publications, e.g., [7,8,19], dealt not only with concentration values of discrete components as reference data, but also with integral parameters such as chemical oxygen demand (COD) and biological (biochemical) oxygen demand (BOD), the measurement of integral water parameters by MSS was underestimated for a long time. While it becomes totally impossible to perform a comprehensive quantitative analysis of all possible water contaminants due to their numerousness, the methods suggesting the global evaluation of water quality open up another realistic approach of water safety evaluation. Similar ideas—integral qualitative recognition and the classification of waters as either safe or dangerous—were once formulated in [20].

The already existing methods providing the global estimation of water quality and its danger or safety for living beings are based on the application of bioassays. The response of different biotests—fishes, crustacea, bacteria, different other microorganisms or even algae—would be induced by most, if not to all, potentially harmful water components. In this approach, it is not necessary to assess individually the content of each component. Thus, it seems reasonable to use such “global” response as reference data for appropriate MSS training.

The application of potentiometric MSS including 23 cross-sensitive sensors for water toxicity estimation in terms of the bioassay was performed in [21]. Three living test organisms—*Daphnia magna*, *Chlorella vulgaris*, and *Paramecium caudatum—*were used for the bioassay procedure. Both model and real water samples were analyzed in this study. The MSS data were analyzed by the PLS regression method; the prediction of water toxicity with relative errors of 15–26% (depending on the microorganisms) was achieved.

A similar to [21] but optimized MSS consisting of 19 sensors was applied for the monitoring of urban waters [22]. The samples were taken in 29 city ponds both from surface and bottom water layers to avoid the influence of stratification. *Daphnia magna* bioassay was used as a reference method. The predictive models built on the MSS data (PLS and PRM (partial robust M-regression) regression) were validated with independent test sets. It was found that the root mean square prediction errors did not exceed 20%. This result can be considered as reliable, since the analytical task was quite complex, and real samples were studied.

Further, an MSS was applied for the safety evaluation of surface waters from different objects [23]. Real samples were taken from a number of ponds, rivers, and lakes in Catalonia, Spain. Fifty-five real and model samples of polluted water were studied. A Microtox^®^ analyzer based on the registration of luminescence from *Vibrio fischeri* bacteria was applied to generate the calibration dataset. The response of MSS comprising 23 sensors was processed with machine learning techniques such as PLS regression, random forest, and K-nearest neighbors. The reported MSS allowed the prediction of toxicity in terms of EC50 (half-maximal effective concentration, or the concentration of sample causing a 50% luminescence reduction) with relative errors of 20–25%.

**Figure 1:**
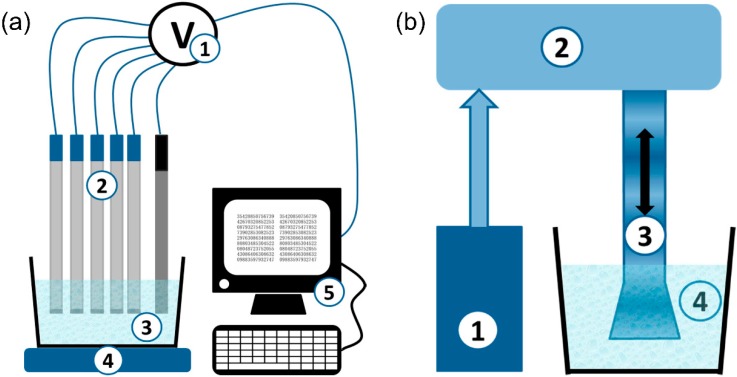
(**a**) Scheme of the potentiometric multisensor system. 1—multichannel digital mV-meter; 2—potentiometric sensors; 3—sample; 4—magnet stirrer; 5—PC. (**b**) Scheme of the UST device. 1—ultrasound generator; 2—magnetostrictive transducer; 3—waveguide; 4—sample [24] © 2019 by the authors.

A potentiometric MSS comprising 20 sensors was recently applied for the rapid evaluation of water quality from rivers, lakes, ponds, and also of wastewater [24] by measuring water toxicity. The toxicity was evaluated both before and after cavitation ultrasound treatment (UST). The MSS setup and a UST device are shown in Figure 1a,b, respectively. Good correlation with bioassay toxicity was found for most samples analyzed before UST.

In addition, this MSS was also used to evaluate the quality of wastewater from two water treatment plants close to St. Petersburg, Russia. The results of the MSS measurements showed a good correlation with the COD values obtained by standard chemical analysis (cross-validation R^2^ = 0.85). The system can also determine some specific water parameters such as ammonium, nitrate’s nitrogen, and phosphorous with the precision about 25%. An elaborated data visualization approach suitable for water treatment process follow-up and purification equipment performance evaluation was also reported in [25].

Other interesting MSS applications for water quality assessment could also be mentioned [26,27,28]. The detection of some typical products of algae decomposition such as 2-methylisoborneol (MIB) and geosmin (GSM) was described in [26] using gold interdigitated microelectrodes coated with ultra-thin polymeric films. MIB and GSM negatively affect the flavor qualities of water and may be toxic at the elevated levels. The MSS identified and quantified these contaminants at the level of 25 ng/L with remarkable reproducibility. Such concentrations of these components are typical for contaminated natural waters.

The assessment of specific cyanotoxins in fresh water by a potentiometric MSS was performed in [27]. The most hazardous microcystin (MC-LR), produced by cyanobacteria and potentially associated with serious health damage, was chosen as an analyte. The MSS consisted of eight sensors: four based on various ionophores and ion-exchangers, three with chalcogenide glass, and one polycrystalline LaF_3_-based sensor. The MSS findings were consistent with the results obtained by a chromatographic technique and colorimetric enzymatic method. Applying PLS regression to the multisensor dataset, the authors achieved RMSEV (root mean square error of validation) values at the level of 1 µ/L, which corresponds to the relevant WHO (World Health Organization) guideline and makes the proposed approach suitable for cost-effective environmental monitoring.

Another MSS for biological toxins assessment in water was proposed in [28]. Paralytic shellfish toxins are neurotoxins produced by various species of marine dinoflagellates. An MSS composed of six miniaturized potentiometric sensors was applied for detecting such toxins in extracts from filter-feeding bivalves. The results were in agreement with chromatographic methods, and MSS may be used for shellfish toxin’s monitoring.

Unusual applications of MSS for the analysis of organic substances or even biologically induced water contaminants, along with possibility of the simultaneous identification and quantification of multiple inorganic analytes, confirm the versatile applicability of such systems for comprehensive water quality analysis. 

## 3. Agricultural Analysis

Soil is the basis of plant growth and development, it supplies the matrix of terrestrial plants, water, fertilizers, and heat required for the normal growth and development of plants. The organic matters in soil provide nutrients, promote the growth of crops, and maintain the activities of soil microbes [29,30]. Artificial fertilization is necessary when the nutrients of the soil cannot satisfy the growth requirements of crops. Precise fertilization control is important in modern agriculture, which needs an accurate detection method of soil nutrients. Detailed soil information is important in precision agriculture management. Soil analysis is also a crucial step in soil mapping and land planning. Precision, speed, cost, and multi-sample processing are important features of modern soil analysis.

Traditional soil analysis methods require sampling and pretreatment steps prior to chemical analysis in the laboratory. Although these detection methods have high accuracy, they are expensive and time-consuming. Moreover, such methods may damage the sample and cause secondary contamination, which motivates the development of sensing in agriculture.

In the recent years, MSS application to soils has been an active and dynamic research field (Table S2, Supplementary Materials). Several electrochemical techniques (voltammetry, electrochemical impedance, potentiometry, and differential pulse polarography) have been used for various applications, for example, the determination of fertigation strategy in greenhouse cultivation [31].

An MSS used in [31] comprised eight potentiometric sensors in the sensitive arrays and complex data processing by ANN. The target ions were the following: NH_4_^+^, K^+^, Na^+^, Cl^−^, and NO_3_^−^. The in-line application of the system in greenhouses showed that the ANN succeeded in compensating the temperature effects, while concentrations of four (besides chloride) of the five studied ions could be correctly predicted.

**Figure 2:**
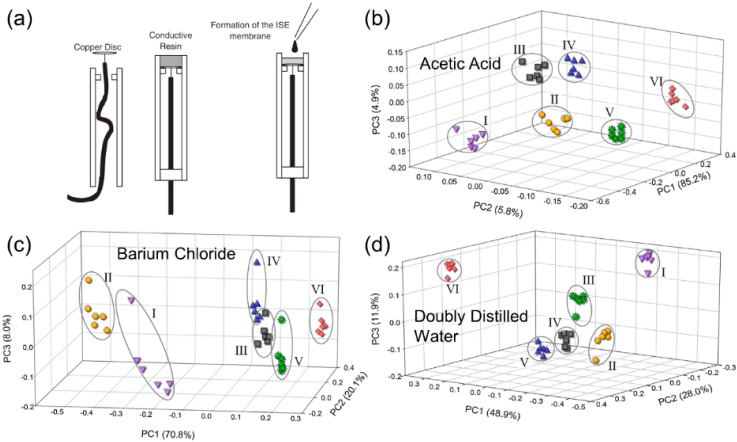
(**a**) Fabrication of ion-selective electrode with electrical solid contact based on epoxy–graphite composite [33] (reprinted with permission of the publisher (Taylor and Francis Ltd.), license 4684171258815); 3D scores plots of the extraction method used (**b**) acetic acid, (**c**) barium chloride, and (**d**) doubly distilled water [32] (reprinted with permission of the publisher (John Wiley and Sons), license 4684171056214).

MSS were also applied for soil discrimination and fertility assessment [32]. The sensor array used in this study consisted of 20 all-solid-state ISEs with polymer membranes. As shown in Figure 2a, the electrodes were made from a conductive epoxy–graphite composite with dropping of the mixture of polyvinyl chloride (PVC), plasticizer, and ionophore [33]. Six kinds of soils from the areas of different climate and parent materials in Catalonia were selected for this study according to their properties such as texture, pH, organic matter, and lime content. A simple process of sample preparation involving liquid extraction, mixing for 1 h by a shaker, and sedimentation for 30 min was developed. The MSS measurements were carried out without any filtering stage. The sensor array was dipped in the sample for at least 5 min to ensure the stability of the potential values. The performance of three extracting agents was evaluated. All the extractions and measurements were replicated six times. The clustering of six soils for each extracting agent is visible in the 3D score plots (Figure 2b–d). Then, the data from each soil were randomly selected and subdivided into training and testing subsets, and an ANN was used as classifier. The results demonstrated satisfactory agreement with the expected distribution of the soil samples.

**Figure 3:**
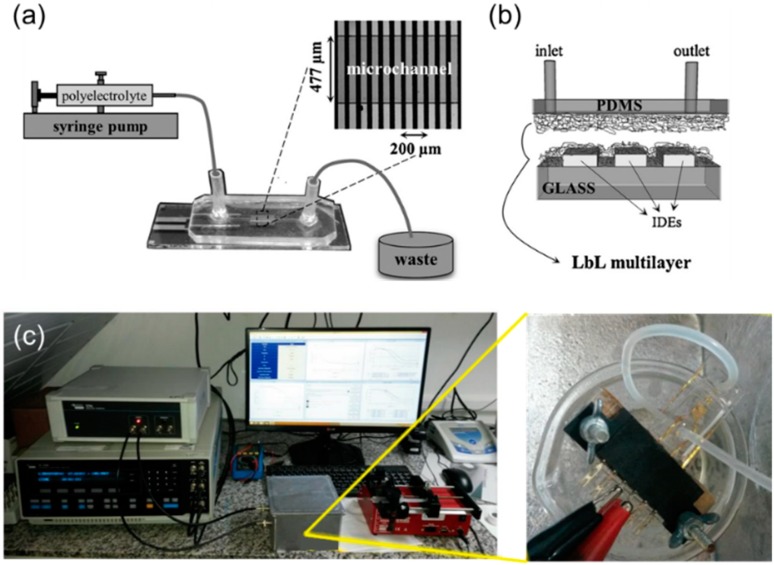
(**a**) Schematic of the system setup used in layer-by-layer (LbL) assembly; (**b**) cross-section view of the LbL film inside the microchannel; reprinted from [35] with permission from Elsevier (license 4684171385345); (**c**) photo of the microfluidic electronic tongue (ET) system [34] © 2017 by the authors.

Recently, MSSs have been incorporated in microfluidic devices due to their benefits of small size, compact structure, less sample volume, and cost reduction. Microfluidic devices integrated with MSS have been already applied to soil analysis [34,35]. The microfluidic MSS used in these works was composed of four sensing units inside a channel made by polydimethylsiloxane (PDMSS). Each sensor unit consisted of 30 pairs of gold interdigitated electrodes (IDEs) covered by layer-by-layer (LbL) films. The microfluidic device is schematically shown in Figure 3. The LbL films were deposited on the IDEs by passing the materials sequentially through the microchannel.

**Figure 4:**
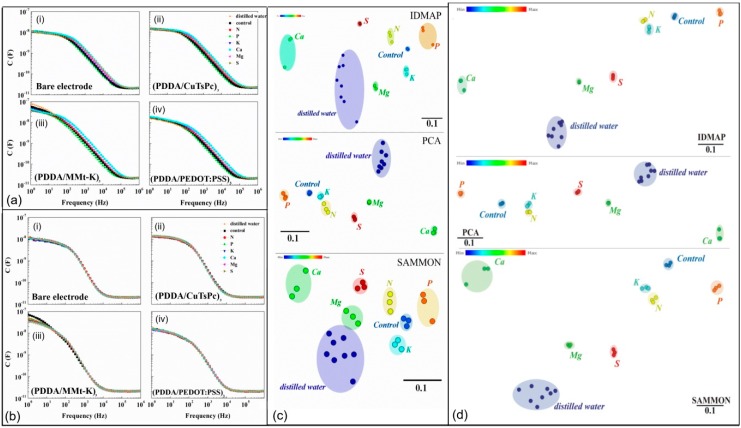
(**a**) Capacitance vs. frequency spectra of seven soil samples (N, P, K, Ca, Mg, S, and control) and (**b**) distilled water after each measurement using (**i**) bare electrode; (**ii**) (PDDA/CuTsPc)_3_; (**iii**) (PDDA/MMt-K)_3_, and (**iv**) (PDDA/PEDOT:PSS)_3_; (**c**) capacitance data using the whole frequency spectra; and (**d**) the selected frequency for the analysis of soil samples [34]. © 2017 by the authors.

To verify the discrimination capacity of microfluidic MSS, soil samples individually enriched with N, P, K, Ca, Mg, and S were prepared. The mixtures were placed in a greenhouse for 40 days to ensure enough chemical reactions between the compounds and the soil. Finally, six soil samples with different elements from those mentioned above were prepared; one blank sample with no extra additive was used as the control sample. Figure 4a shows the capacitance spectra of the four sensors of the MSS exposed to the seven soil sample solutions. As shown in Figure 4b, the tiny displacements indicate no cross-contamination, which confirms the sensor reusability. The normalized soil data of the whole spectra (1 Hz to 1 MHz) were analyzed by PCA, interactive document map (IDMAP), and Sammon’s mapping (SAMMON); the results are shown in Figure 4c. Furthermore, the parallel coordinates technique was applied to optimize the frequency range from 79 Hz to 25 kHz, and the results within the selected frequency range are shown in Figure 4d. The distance between the data points of the same class is significantly smaller than the distance between the different classes, which ensures the easy distinction of the soil samples with various additives.

A microchip capillary electrophoresis MSS for soil nutrient analysis was reported [36]; the sensor system is sensitive to NO_3_, NH_4_, K^+^, and PO_4_, which is important for plant nutrient monitoring. The sensor consisted of a microfluidic chip where the sample ions are separated in an electric field (capillary electrophoresis) and the individual ion concentrations are detected by a conductivity measurement. The system was tested on real samples. It was found that nitrate and potassium ions could be detected, while the DL for ammonium and phosphates were too high for practical analysis. Although the proposed system is not a real MSS example, the authors applied a rather similar methodology of multichannel measurements.

**Figure 5:**
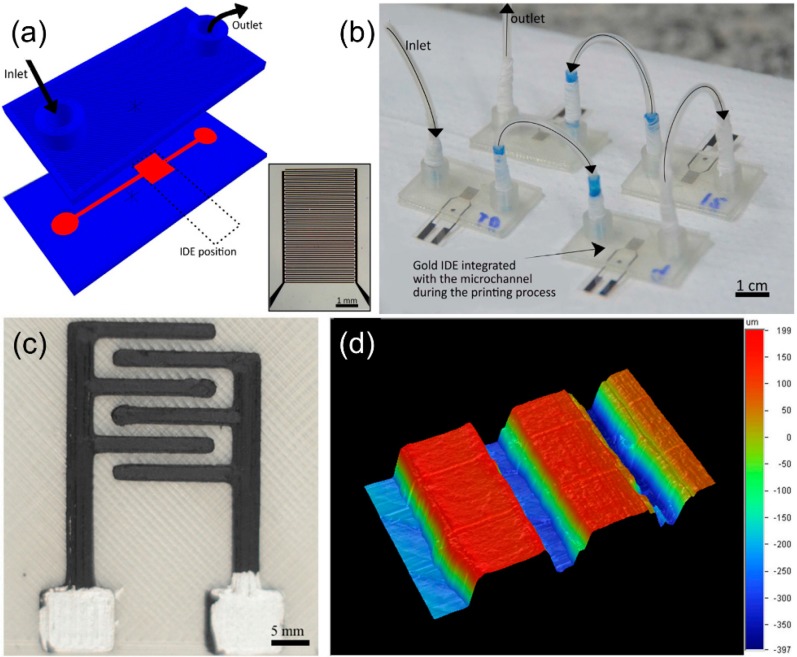
(**a**) Three-dimensional (3D) model of the electronic tongue sensor (the microchannel is in red). The interdigitated electrodes (IDEs) were manually inserted during the printing process; (**b**) photo of the microfluidic electronic tongue system ((a) and (b) reprinted from [37] with permission from Elsevier (license 4684171493577)); (**c**) 3D printed IDEs with three pairs of fingers 9 mm long, 1 mm wide, and intervals with 0.4 mm thickness; (**d**) 3D profilometry mapping of the printed IDE to estimate the root mean square (RMSS) surface roughness [38]. © 2018 Gaál, da Silva, Gaál, Hensel, Amaral, Rodrigues, and Riul.

A number of MSS studies related to agricultural applications concern the 3D printing technology. A polylactic acid (PLA) microchannel was 3D printed and combined with the IDEs deposited on transparent sheet (Figure 5a,b) [37]. The 3D printing of this microfluidic MSS takes only an hour, which is difficult to achieve with conventional polydimethylsiloxane materials requiring casting, curing, and bonding. Recently, the IDEs were successfully fabricated within 6 min by 3D printing using transparent graphene-based PLA filaments (Figure 5c,d) [38] and have been applied in soil analysis to discriminate soils enriched with different nutrients. The results show that 3D printing technology enables rapid prototyping with greater design flexibility and paves the way for future developments.

Certainly, one of the most actual areas of MSS application not only in agriculture but in general practice is the detection of pesticides.

A widespread use of pesticides in agricultural activities can cause serious health and environment issues, since these compounds can be highly toxic. Thus, novel technologies that are capable of monitoring pesticides at very low concentration levels are of outmost importance. The research and development (R&D) of sensors and biosensors in this field is highly demanded, including MSS demonstrating high sensitivity and a low limit of detection for in situ measurements. Remarkable advances have been made in the last decade in the development of MSS detecting different types of pesticides.

A series of works [39,40,41] was devoted to the development of low-cost chips based on screen-printed biosensors and ANN for the detection of pesticides. The selective quantification of chlorpyrifos oxon (CPO) and chlorfenvinfos (CFV) in their mixtures by an array of AChE biosensors and ANN using a PIC (Programmable Interface Controllers) microcontroller was reported in [39]. The developed low-budget system was able to accurately quantify the concentration of pesticides with a low error level.

A few years later, the same authors reported [40] the development of disposable biosensors based on two genetically modified enzymes and ANN for analysis of the mixture of three organophosphorus (OP) compounds, i.e., CPO, CFV, and azinphos-methyl oxon (AZMO), through the calculation of an irreversible enzyme inactivation rate (kp). This study demonstrated successful results, although the number of samples for ANN training was still not sufficient for some persuasive conclusions.

Furthermore, in [41], these authors used a similar MSS system to detect paraoxon, dichorlvos, and carbofuran pesticides. They also immobilized different AChEs onto screen-printed carbon electrodes (SPCE) electrodes and used Ag/AgCl as a reference electrode, exploring the inhibition process to both detect and quantify the presence of OP and carbamate pesticides. Unlike [41], the authors proposed pralidoxime (2-pyridine aldoxime methyl chloride, 2-PAM) for the reaction with AChEs, along with an automated pumping system for on-line pesticides monitoring.

In [42], the AChEs inhibition process in the presence of OP pesticides was also studied using electrochemical measurements. It is known that these pesticides inhibit the AChEs, converting AChEs into choline, which is further reduced to H_2_O_2_, and S-acethylcholinesterase into thiocholine. The electrode geometry based on SPCE and a reference Ag/AgCl reference electrode was developed. The modification of carbon ink with cobalt phthalocyanine (CoPC) was performed, allowing the detection of electroactive thiocholine. The thiol group reduces the Co^2+^ to Co^+^, the latter is further re-oxidized to Co^2+^ at 0 V versus Ag/AgCl. As a result, the inhibition of AChEs reduces the production of thiocholine, reducing the measured electrochemical current. Each AChE suffers from different inhibition processes; therefore, combining an array of AChEs with hierarchical cluster analysis (HCA) and PCA classification methods, the authors were able to determine OP pesticides in the range from 10^−5^ to 10^−9^ M. Although the research outline in [42] was profound and detailed, the number of analytes was too small for reliable conclusions.

It was shown in [43] that N-methyl carbamate pesticides decompose into specific reactive phenol groups when they are pretreated with a strong basic solution. As in [43], the indirect method to detect the presence of OP or carbamate pesticides in the solutions was used. A colorimetric sensor array was used to detect the presence of H_2_O_2_ and thiocholine in the samples, checking for traces of OP or carbamate pesticides. Then, the less the amount of H_2_O_2_ and thiocholine content in the sample, the more decreased the colorimetric sensitive indicator intensity. Using HCA and PCA analysis, the authors demonstrated the successful differentiation of OP and carbamates from each other and from other pesticides. However, the lowest experimentally determined concentrations of pesticides were at the level of 10^−7^ g/L, while the LOD values, being the most common analytical figures of merit, were only calculated (around 10^−8^ g/L).

It was already mentioned (in the water analysis section) that the idea to use biosensors in the sensor arrays might seem to be controversial. Although ANN or cluster analysis methods, applied in [40,41,42,43], helped to improve the results obtained from certain biosensors, the reported works pertain mostly to OP and carbamate pesticides, while the diversity of potentially dangerous substances of various classes is much wider. According to the published results, AChEs used in all the biosensors mentioned above would be first of all sensitive to OP pesticides. It is not clear if the biosensors with increased cross-sensitivity to different classes of pesticides can be ever developed and practically applied.

Different enzyme-free sensors and corresponding MSS were also suggested for pesticide detection. A low-cost MSS comprising graphite interdigitated polyethylene terephthalate substrates modified with electrospun nanofibers of polyamide 6 coated with LbL films of polypyrrole (PPy) and poly(o-ethoxyaniline) (POEA) was suggested in [44]. In this work, the MSS was included into a flow analysis system that was capable of discriminating paraoxon (widely used pesticide in corn crop activities) in water samples down to ppb (parts per billion) levels. However, quantitative analysis was not performed.

Atrazine, a highly toxic pesticide, was detected in [45] down to picomolar concentration using surface-enhanced Raman scattering (SERS), since atrazine has a relatively strong Raman signal. The pesticide was incorporated into colloidal silver nanoparticles (AgNPs), and the dataset was treated with an information visualization technique (SAMMON). Ultra-thin films assembled by the LbL technique with gold and silver nanoparticles in their structure were also assembled on Pt interdigitated electrodes used as sensing units for impedimetric and voltammetric e-tongue setups. The lowest detected atrazine concentrations were at 10^−9^ M for voltammetry, 10^−10^ M for impedance measurements, and 10^−12^ M for SERS. Despite the good sensitivity achieved with impedance measurements, the authors noticed that the IDEs were irreversibly affected during electrochemical measurements that did not occur in SERS experiments.

A fluorescent MSS for the simple and fast detection of pesticides at the ppb level was suggested in [46]. The sensor allowed distinct spectra fingerprints of four pesticides, and the use of ANN provided high accuracy and precision in the detection and discrimination of carbamate (aldicarb), organochlorine (chlorothalonil), pyrethroid (deltamethrin), and OP (fenitrothion) pesticides. A large number of training samples was used for multivariate model building; however, real samples were not studied. The additional tests of the applicability of the proposed MSS for practical tasks are required.

The influence of pesticides on the aggregation process of citrate-capped gold nanoparticles (AuNP) was utilized as the source of the analytical signal in [47]. The materials forming the sensor array were processed at different ionic strengths to influence directly the aggregation process with pesticides, thus changing the observed absorbance spectrum of the AuNPs. Consequently, each sensing unit was supposed to exhibit a unique response to each pesticide, enabling discrimination through HCA and PCA analysis and the quantification (by linear discriminant analysis, LDA) of OP at hundreds of ng/mL. Unfortunately, a few samples studied in this research would not allow unambiguous conclusions about perspectives of the suggested method.

An optical MSS to identify pesticides, imidacloprid and paraoxon, was studied in [15]. The authors used a combination of 10 sensing units comprised of pH indicators, porphyrins, and their blends mixed with anionic and cationic ions exchanger salts. The interaction between the analyte and sensing units provides a unique absorption pattern under the illumination of RGB LEDs (Red Green Blue Light-Emitting Diodes). The authors were able to classify the observed patterns just measuring the light intensity, determining the presence of the pesticides and quantitatively correlating the red and green channels intensity with the concentration of the pesticides below µM.

In recent decades, impedimetric MSS has been also proposed for the development of enzyme-free pesticide sensors. The development of an enzyme-free impedimetric MSS that is capable of detecting OP pesticides at 0.1 nmol L^−1^ concentration was reported in [48]. Malathion and cadusafos pesticides, which are widely used in agricultural activities, were also chosen as analytes. The authors modified four gold IDEs using hybrid nanocomposites composed of reduced graphene oxide, poly(3,4-ethylenedioxythiophene)/poly(styrenesulfonate) (PEDOT:PSS), PPy, and AuNPs. The four sensing units of the e-tongue system were composed by IDEs modified by drop casting with the hybrid nanomaterials. The raw data obtained by impedance spectroscopy measurements were analyzed by PCA. The results showed that the system was able to discriminate OP at nanomolar concentrations in distilled and tap waters. However, the quantitative results can be considered only as preliminary due to the small number of the samples under study.

Recently, a colorimetric MSS was also proposed as a beneficial tool for the fast and easy detection of pesticides [49]. A simple colorimetric sensor array based on ensembles of sulfuric acid and potassium permanganate (KMnO_4_), which can be fabricated by properly adjusting the concentrations and ratios between both compounds, was applied. The recognition and discrimination of various pesticides (including metolcarb, carbaryl, isoprocarb, deltamethrin, permethrin, cypermethrin, chlorpyrifos, diazinon, triazophos, glyphosate, phoxim, methomyl, DDT (Dichlorodiphenyltrichloroethane), α-BHC (α-Benzenehexachloride), pretilachlor, and MCPA (2-methyl-4-chlorophenoxyacetic acid)) was carried out using HCA. The semi-quantitative pesticides detection was achieved by the combination of HCA with corresponding fitting curves, and the proposed colorimetric sensor was also capable of detecting pesticides in real samples.

A cheap, fast, and reusable microfluidic-based fluorescent sensor array was suggested to detect pesticide residues (carbendazim, diazine, fenvalerate, and pentachloronitrobenzene) with high sensitivity [50]. The system can give new opportunities for MSS devices, as a fingerprint-like response can be obtained from colorimetric patterns. Macroscopic characteristics of the samples can be extracted using unsupervised computational methods, with the MSS sensitivity down to the ppb level.

A very recent paper [51] deals with the development of a simple and enzyme-free colorimetric sensor for rapid pesticides identification using silver nanoparticles (AgNPs). The AgNp colorimetric sensor array was specifically used for the simultaneous determination of azinphosmethyl (AM) and phosalone (PS) pesticides through the distinct aggregations of AgNps that yielded detectable color changes. Using linear discriminant analysis (LDA), the proposed sensor array performance was evaluated by identifying the target pesticides in apples. Although the reported results are encouraging, only two pesticides were chosen as analytes, and it is difficult to conclude if the developed MSS can be used for the detection of other potentially toxic substances.

## 4. Medical Analysis

**Figure 6:**
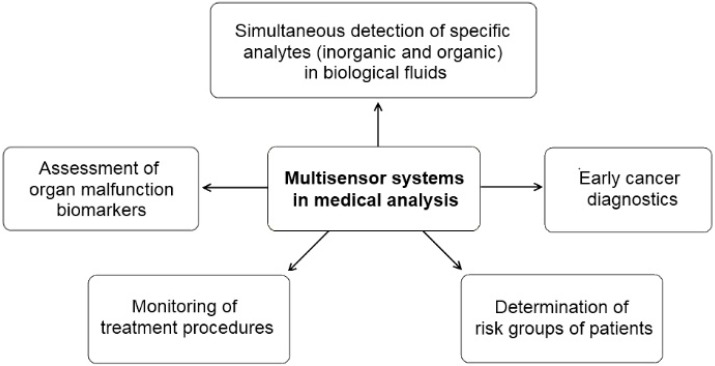
Application of multisensor systems in medical analysis.

Blood, urine, tears, sweat, and saliva are important biological fluids that provide information about the possible health issues of a patient. Human expert panels are not suitable for the analysis of such samples, especially in cases when pathological conditions or metabolic disorders are investigated. These fluids are mainly composed of water and a small amount of biomarkers such as proteins, amino acids, and various organic and inorganic compounds [52]. The MSS discussed in this review are promising tools for the monitoring of such biomarkers, since they work in complex liquid media. The results of some studies of MSS performance in clinical analysis are summarized in Table S3 (Supplementary Materials). The main medical applications of MSS are presented in Figure 6.

An MSS comprising an array of 30 non-specific chemical sensors and BPNN and PLS as data-processing algorithms was applied in [53] for the analysis of multicomponent solutions modeling the biological liquids. It was found that this approach allows the simultaneous determination of Ca^2+^, Mg^2+^, HCO_3_^2−^, H^+^, and HPO_4_^2−^ ions in concentration ranges typical for human blood plasma with an average precision of 2 ± 4%, which is relevant for clinical analysis. However, these results should be verified in the experiment with real biological liquids.

The fusion of gas (electronic nose, EN) and liquid (potentiometric electronic tongue, ET) MSSs composed of the metalloporphyrin-based sensor array enabled the evaluation of urine samples collected from children affected by kidney diseases and healthy ones [54]. The pH, specific gravity, and presence of blood were assessed in the urine samples with a commercial urine test kit used to validate the results. Pattern recognition of the samples was performed by PCA, which revealed a better correlation between pH and the specific gravity for the data obtained by the e-tongue system (61.3–91.4% and 55.7–79.9%, respectively), while the amount of blood cells was correlated mostly with the findings of the e-nose analysis (90–98.6%). The MSS performance was improved due to the increased amount of information extracted from the samples, which is a result of the sensorial fusion of ET performing in liquid and EN working in the headspace [55].

Urea is an important biomarker to assess kidney malfunction and to control dialysis efficiency. Usually, the electrodes selective to urease are applied for its detection in serum and urine samples. However, their sensitivity is usually affected by the presence of ammonium, potassium, and sodium ions. In this regard, Gutiérrez et al. proposed a bio-electronic tongue comprised of biosensor arrays of urea and ion-selective electrodes (12 in total) with urease immobilized onto two ammonium and two hydrogen electrodes, aiming for the simultaneous detection of urea and its most severe interferences in urine samples diluted and spiked with distinct analytes [56]. Due to the high non-linearity of the potentiometric response, ANN methods were more efficient for the ET data processing than linear algorithms such as PLS, with typical individual errors of 8% for ANN and 13% for PLS. The average recovery values for urea and ammonium were 78% and 90%, respectively, according to the reference values determined by conventional Berthelot reaction for urea, inductively coupled plasma optical emission spectrometry (ICP-OES) for potassium and sodium, and ammonia gas electrodes. This simple procedure enabled the determination of urea concentration in real samples without of the necessity of compensation of endogenous ammonia. However, it should be mentioned that the errors of 8–13% can be rather high for clinical analysis.

One year later, the same research group studied creatinine, which is another important biomarker of renal dysfunction [57]. In fact, urea and creatinine clearance are generally assessed within the early diagnostics of kidney diseases. A sensor array comprised of urea biosensors, creatinine biosensors, ISEs for ammonium, potassium, sodium, and electrodes of generic response to alkaline ions (two units of each kind, 12 electrodes in total) was used in bio-electronic tongue for urine samples analysis. The potentiometric response was successfully processed by ANN, exhibiting a low (1.8 mM) root mean squared error (RMSE) and average recoveries of 92.6%, 101.4%, 101.3%, 108.7%, and 95.8% for urea, creatinine, ammonium, potassium, and sodium, respectively. It should be noted that the results for about one-quarter of the test samples were obtained with significant errors.

Dialysis is a life-sustaining procedure for patients with renal failure; an artificial kidney (hemodialyzer) is used to remove waste products (for example potassium, acid, creatinine, and urea) from blood and also to eliminate excessive fluid in the form of urine. Since dialysate fluids have significantly less complex matrix than blood or urine, it would be advantageous to develop a measurement procedure that would not need any sample pretreatment. A potentiometric MSS based on ISEs and biosensors was applied to detect creatinine and urea in dialysate fluids [58]. The system response was processed by PLS; it was possible to differentiate the samples containing high and low levels of analytes. According to the authors, the method is more beneficial than the existing techniques (urine and blood analysis) and can be applied for the continuous monitoring of dialysis procedures.

The possibility of discriminating biological fluids affected by malignant tumors was proposed in [59]. In this study, potentiometric MSS comprised of metallic sensors and PVC-based membrane electrodes were firstly applied for urine samples classification according to the creatinine level. Using partial least squares discriminant analysis (PLS-DA), the authors achieved 84.31% of correctly classified samples. The result was further improved through feed forward back-propagation ANN, achieving the accuracy of 92.16% compared to Jaffe’s method and gas chromatography-mass spectrometry (GC-MS). Then, the performance of this MSS was evaluated in the classification of urine samples from patients with bladder tumors and healthy volunteers. The urinary samples were collected from 17 patients with bladder tumors (16 malignant and one non-malignant tumor) and 10 healthy volunteers for control and analyzed via PCA for simple pattern recognition. The dissimilarities between the samples from the patients and controls were clear, since cancer presence may change the chemical composition of urine. However, more statistically reliable proof is needed to apply this method in clinical practice.

Blood serum samples from patients with glaucoma and presumably healthy individuals were assessed by both liquid and gas-sensing MSS [60]. For EN, LDA analysis resulted in the correct classification of 10 out of 15 samples from the control group (67%) and 21 out of 27 glaucoma patients (77%). Liquid MSS could distinguish only three out of six control serum samples (50%) and nine out of 14 glaucoma patients (64%). The low effectiveness of both methods can be explained by the use of non-modified SPCE, since it is nearly impossible to detect the certain biomarkers in complex biological fluid samples using a non-specific MSS.

One of the main indicators of a patient’s health condition is urine ionic composition. A potentiometric MSS was used to determine the content of different ions normally presented in the urine [61]. The MSS was composed of 19 sensors with membranes based on anion- and cation-sensitive ionophores and anion exchangers. The analysis of 136 urine samples (one per patient) demonstrated that the results were comparable to the accuracy of capillary electrophoresis. PLS was used for the prediction of the urine components concentration with mean relative errors below 10% for sodium, ammonium, and chloride, and below 15% for potassium, calcium, magnesium, sulfate, phosphate, urate, and creatinine. According to the obtained results, the MSS is a promising alternative method for urolithiasis diagnostics.

Sweat is also an important body fluid for clinical analysis, as variations in its ionic composition can be related to exercise, diet, or health issues. In this context, a potentiometric MSS was applied to evaluate the concentration of Na^+^, K^+^, and Ca^2+^ in synthetic and real human sweat samples [62]. The sensing units were based on solid-contact technology and were compared to conventional potentiometric ISEs. The MSS results were compared with the data obtained by flame photometry and atomic absorption spectrometry (AAS). The MSS response was reproducible; however, the ionic concentration of sodium (6.68–8.77%) and potassium (7.07–10.68%) were lower than those obtained by flame photometry, and the Ca^2+^ concentration measured by MSS was higher than the AAS results (16.44–22.37%).

Another potentiometric MSS based on ion-sensitive field-effect transistors (ISFETs) and extended gate field-effect transistors (EGFETSs) was applied to predict urinary stone formation by measuring Ca^2+^ and pH in urine samples [63]. The variations of calcium levels in urine samples can indicate some health disorders, such as kidney stone formation. Unfortunately, since no comparative figures of merits characterizing the MSS performance were presented, it is impossible to make some conclusions about its applicability for clinical tasks.

A voltammetric MSS comprising seven working electrodes (Ir, Rh, Pt, Au, Ag, Co, and Cu) versus saturated calomel reference electrodes was applied to detect patients with prostate cancer (PCa) [64]. The sample set comprised 114 samples: 71 samples from the patients with PCa before surgical treatment and 43 controls (26 from patients who underwent the surgery and 17 from individuals with benign prostatic hyperplasia). Supervised PLS-DA was able to correctly classify 20 Pca samples from 22 and only 11 out of 15 controls. This method outperformed the conventional prostate-specific antigen (PSA) blood test with a high sensitivity of 91% and specificity of 73%.

Elevated protein levels in urine can be caused by several health issues. The increased protein level can be temporary or persistent depending on the cause, e.g., short-term dehydration and diabetes, respectively. A continuously evolving colorimetric MSS for protein discrimination was suggested in [65]. The sensor was based on AuNPs modifed with two single-stranded oligonucleotides with variable molar ratios. Then, the colorimetric patterns caused by the AuNPs aggregation were evaluated by LDA. The system was able to differentiate pure proteins, such as concanavalin A (Con-A), cytochrome C (Cyt-C), egg white albumin (EA), hemoglobin (Hem), horseradish peroxidase (HRP), human serum albumin (HSA), immunoglobulin G (IgG), lysozyme (Lys), myoglobin (Myo), pepsin (Pep), transferrin (TRF), and trypsin (Try) and the mixtures of Lys/Try, Lys/Pep, and Lys/HSA. All the proteins spiked in urine samples were easily identified, showing a high potential of the colorimetric MSS for protein components determination in clinical tests.

Many techniques for urinary creatinine content evaluation require sample dilution with other reagents to overcome the obstacles caused by the matrix effect. A voltammetric MSS to determine the creatinine content in 59 urine non-diluted samples was proposed in [66]. The MSS comprised seven working electrodes (glassy carbon, Au, Pt, Ag, Ni, Pd, and Cu), Ag/AgCl as a reference, and Pt as the auxiliary electrode. The results obtained by cyclic voltammetry were analyzed by PCA and support vector machines (SVM) in order to classify the urine samples according to low, medium, and high levels of creatinine. In addition, the authors used Jaffé’s reaction as a reference colorimetric method, obtaining high correlation coefficients with PLS regression. Nevertheless, no independent test set was used to check the system’s performance, and the reasons for some obvious outliers among samples were not properly explained.

An MSS consisting of 28 potentiometric sensors (PVC plasticized with chalcogenide glass and polycrystalline membranes) was used in the analysis of urine from 43 prostate cancer patients and 46 healthy ones from a control group [67]. Various data-processing methods were applied: PCA, soft independent modeling of class analogy (SIMCA), PLS-DA, logistic regression (LR), and random forest (RF). The best classification model was obtained with logistic regression (LR), showing 100% sensitivity and 93% specificity. In spite of the very promising first results, further work remains to be done for the thorough validation of the approach, especially from the point of view of the experimental design and number of samples, including the problem of the time distribution of samples and multivariate calibration sustainability.

Some more challenging directions of “inedible” MSS research, such as the analysis of complex industrial mixtures (e.g., [68]) are also of great interest, but it seems a little too early to review these emerging areas.

## 5. Conclusions

The diverse scientific papers devoted to the MSS application for the analysis of inedible samples were discussed in this review. Certain applications, e.g., the analysis of natural and waste waters, pesticides in agriculture, and different medical analytes, are quite mature, and the number of papers published on these topics is constantly increasing, along with the complexity and importance of the tasks explored by the authors. After more than 20 years of extensive scientific research, the “electronic tongues” are likely to be entering the era of advancements in certain practical fields. Some of these fields may be rather different from those that one could imagine 20 years back, but it proves the validity of ideas standing behind such systems and the decent quality of research performed by many scientific teams in many countries.

## Supplementary Materials

The following are available online at https://www.mdpi.com/1424-8220/19/23/5113/s1, Table S1: Summary of the studies concerned with multisensor systems (MSS) application for water analysis. Table S2: Summary of the studies concerned with MSS application for agricultural analysis., Table S3: Summary of the studies devoted to the application of MSS for medical analysis.
