# Supplementary material for: Electronic Tongues for Inedible Media
Source: Sensors (Basel). 2019 Nov 22;19(23):5113. doi: 10.3390/s19235113 (PMC6928786; doi:10.3390/s19235113)
Supplement: Supplementary file 1 [file sensors-19-05113-s001.pdf]

Supplementary Materials

Table S1. Summary of the studies concerned with multisensor systems (MSS) application for water analysis.

Target Analytes/ Integral Characteristics	Sensor Type	Concentration Range/ Groups of Samples	Reference
Heavy metal cations and inorganic anions	Ion-selective electrodes	From 1 to 10^{-5} mmol/L depending on the analyte	[2,3,14]
Inorganic anions (cyanide)	Potentiometric sensors	10–100 ppm	[4]
Purification rate	Voltammetric sensors	Water samples after different filters	[5,19]
Mineral water brand	Impedimetric sensors	Several water brands and tap water	[6]
	Potentiometric sensors		[17,18]
Biochemical oxygen demand and PAH *-degrading ability	Amperometric biosensors	1–50 mg/L	[7]
Quality of water treatment	Enzymatically-modified amperometric sensors	Four water treatment levels	[8]
	Potentiometric sensors	Samples from two wastewater treatments plants	[25]
Pesticides	Enzymatically-modified amperometric sensors	0–16 nmol/L	[9,10]
Soil nutrients	Ion-selective electrodes	From 0.1 to 700 mg/L depending on the analyte	[11,12]
Nitrogen-containing inorganic compounds	Ion-selective electrodes	0.01–20 mmol/L	[13]
Hazardous substances	Optical sensors	10^{-4}–10^{-7} mol/L	[15]
Toxicity assessment	Potentiometric sensors	Various figures of merit in terms of bioassay	[21–24]
Contamination by biological toxins	Impedimetric sensors	25–1000 ng/L	[26]

	Potentiometric sensors	0–300 mg/L	[27]
	Potentiometric sensors	0.2–7.2 μmol/L	[28]

* PAH: polycyclic aromatic hydrocarbons.

Table S2. Summary of the studies concerned with MSS application for agricultural analysis.

**Target Analytes/ Integral Characteristics**	**Sensor Type**	**Concentration Range/ Groups of Samples**	**Reference**
Inorganic ions	Ion-selective electrodes	From 0.7 to 20 mmol/L depending on the analyte	[31]
	Microchip capillary electrophoresis device	5–500 μmol/L	[36]
Soil type and fertility assessment	Ion-selective electrodes	Six types of soil	[32]
	Microfluidic interdigitated electrodes	Several types of enriched soils	[34,35,37,38]
Pesticides	Enzyme biosensors	10^{-7}–10^{-10} mol/L	[39–43]
	Interdigitated electrodes	1–10 ppb	[44,45]
	Fluorescent sensors	10–50 ppb	[46,50]
	Nanoparticle-based colorimetric sensors	100–4000 ng/mL	[47,51]
	Colorimetric sensors	10^{-5}–10^{-6} g/L	[15]
		10^{-4}–10^{-6} mol/L	[49]
	Impedimetric sensors	0.1–5 nmol/L	[48]

Table S3. Summary of the studies devoted to the application of MSS for medical analysis.

Analyte	Samples	Sensing units	Principle of detection	Limit of detection	Concentration range	Computational analysis	Ref.
Ca2+, Mg2+, Na+, HCO32-, Cl-, H+, HPO42- ions	45 solutions modelling human blood plasma	19 soli state and polymeric chemical sensors	potentiometry	-	0.03-150 mmol/L, typical for each component in blood plasma	PLS, ANN	[56]
ascorbic acid, uric acid and acetaminophen (paracetamol)	50 standard solutions prepared by SIA	platinum, gold and epoxy-graphite discs	linear sweep voltammetry	-	0.1 – 1.1 mM	ANN	[57]
urea, ammonium, potassium and sodium	diluted and spiked urine samples	urease immobilized onto PVC-COOH layer of ammonium and hydrogen ISEs; ammonium, potassium, sodium, hydrogen and generic ISEs	potentiometry	-	10-4 – 10-2 M (urea); 5x10-6 – 5x10-4 M (ammonium); 5x10-5 – 5x10-3 M (potassium and sodium)	ANN; PLS	[58]
urea, creatinine, ammonium, potassium and sodium	raw and spiked urine samples	urease and creatinine deiminase immobilized onto ammonium ISEs; ammonium,	potentiometry	1.0x10-4 M (creatinine)	0.1 - 10 mM (urea and creatinine); 0.005 - 0.5 mM (ammonium); 0.005 - 0.5 mM	ANN	[59]

		potassium, sodium and generic ISEs			(potassium and sodium)		
creatinine	59 urine samples without dilution	7 working electrodes: glassy carbon, Au, Pt, Ag, Ni, Pd, Cu.	cyclic voltammetry	-	2 – 30 mmolL-1	PCA; SVMs; PLS	[66]
sodium, potassium, ammonium, calcium, magnesium, chloride, sulfate, phosphate, urate and creatinine	136 urine samples	19 chemical sensors: Na+, K+, NH4+, Ca2+, Mg2+, Ca2+ + Mg2+, NO3-, Cl-, CO32-, SO42- ; 5 PVC-plasticized anion-sensitive based on various anion-exchangers; 3 PVC-plasticized cation-sensitive based on different tetraphenyl borate derivatives; pH electrode	potentiometry	-	mM	PCA; LR; PLS	[61]
Ca2+ (kidney stones)	urine samples	12 ISFETs; 5 EGFETs based on thin film of SnO2/ITO/Glass and the immobilized Ca2+ ionophore on PVC	potentiometry	-	0.1 mmolL-1 – 1 molL-1	LR; HSPICE	[63]
Na+, K+, Ca2+	synthetic and real human sweat samples	ISEs based on PVC layers; ISEs with solid reference electrode based on PVA	potentiometry	-	mM	-	[62]
urea and creatinine	dialysate fluids with creatinine and urea additives	ISEs based on PVC and different amounts of lipophilic additives; ISEs based on PVC-COOH modified by	potentiometry	-	3 – 12 mmolL-1 (urea); 0.10 – 0.32 mmolL-1	PLS	[58]

		urease and creatinine deiminase; pH electrode					
Concanavalin A (Con-A), Cytochrome C (Cyt-C), Egg white albumin (EA), Hemoglobin (Hem), Horseradish peroxidase (HRP), Human serum albumin (HSA), Immunglobulin G (IgG), Lysozyme (Lys), Myoglobin (Myo), Pepsin (Pep), Transferrin (TRF), Trypsin (Try), mixture of proteins: Lys/Try, Lys/Pep and Lys/HAS	target proteins in aqueous solutions; diluted urine samples spiked with target proteins	AuNPs decorated by two single-stranded oligonucleotides (TBA and T15) with different molar ratios	colorimetry	200 nM	50 – 1000 nM	LDA	[65]
creatinine (bladder tumor)	51 urine samples	miniaturized metallic sensors (metal of high purity and alloys) and ISEs with PVC solvent polymeric membranes	potentiometry	-	10-5 - 10-1 M	PCA; PLS-DA; FFBP NN	[59]
prostate cancer	114 urine samples (71 pre-surgery, 26 post-surgery and 17 with benign prostatic hyperplasia)	7 working electrodes: Ir, Rh, Pt, Au, Ag, Co, Cu	large amplitude pulse voltammetry	-	-	PLS-DA	[64]

prostate cancer	89 diluted urine samples (43 cancer patients and 46 in control group)	28 sensor membranes based on PVC-plasticized, chalcogenide glass and polycrystalline materials	potentiometry	-	-	PCA; SIMCA; PLS-DA; LR; RF	[67]
glaucoma	20 blood-serum samples (14 sick and 6 healthy humans)	screen printed carbon electrodes	inversion voltammetry	-	-	LDA	[60]

Abbreviations/acronyms: artificial neural networks (ANN); carboxylated poly(vinyl chloride) (PVC-COOH); cyclic voltammetry (CV); extended gate field-effect transistor (EGFET); feed forward back-propagation neural network (FFBP NN); gold nanoparticle (AuNP); Hailey - simulation program with integrated circuit emphasis (HSPICE); ion-selective electrode (ISE); ion-sensitive field-effect transistor (ISFET); linear discriminant analysis (LDA); logistic regression (LR); partial least squares (PLS); partial least squares discriminant analysis (PLS-DA); polyvinyl acetate (PVA); poly(vinyl chloride) (PVC); principal component analysis (PCA); random forest (RF); sequential injection analysis (SIA); soft independent modelling of class analogy (SIMCA); support vector machines (SVMs).
